# Prevention of incisional hernia with biosynthetic mesh at the site of temporary ileostomy closure (PRINCESS Study): Preliminary Results

**DOI:** 10.1007/s10029-025-03462-0

**Published:** 2025-09-18

**Authors:** Roberto Peltrini, Carla Rognoni, Giovanni Bellanova, Marco Caricato, Massimo Carlini, Stefania Cimbanassi, Francesco Corcione, Federico Cozzani, Diego Cuccurullo, Giuseppe Faillace, Silvia Neri, Alberto Patriti, Mauro Santarelli, Vincenzo Trapani, Gabriella Teresa Capolupo, Gabriele Carbone, Desiree Cianflocca, Stefano Cioffi, Maria Michela Di Nuzzo, Davide Ferrara, Paola Antonella Greco, Biancamaria Iacone, Francesca Pecchini, Matteo Rossini, Michele Sacco, Stefano Sala, Domenico Spoletini, Salvatore Tramontano, Rosanna Tarricone, Giampiero Campanelli, Giuseppe Cavallaro, Micaela Piccoli, Giorgio Soliani, Cesare Stabilini, Umberto Bracale

**Affiliations:** 1 Department of Public Health, University of Naples Federico II, Via Pansini 5, Naples, 80131 Italy; 2 Centre for Research on Health and Social Care Management (CERGAS), School of Management, SDA Bocconi, Bocconi University, Via Sarfatti 10, Milan, 20136 Italy; 3 UOC Chirurgia Generale, “D. Camberlingo” Hospital, Francavilla Fontana, Italy; 4 UOC Chirurgia Colorettale, Fondazione Policlinico Universitario Campus Bio-Medico Di Roma, Via Alvaro del Portillo, Rome, 21 - 00128 Italy; 5 Department of General Surgery, General Surgery Unit, S. Eugenio Hospital, Rome, Italy; 6 General Surgery and Trauma Team, GOM Niguarda, Piazza Ospedale Maggiore 3, Milan, 20162 Italy; 7 Clinica Mediterranea, Via Orazio, 2, Naples, 80122 Italy; 8 General Surgery Unit, University Hospital Parma, Parma, Italy; 9 Department of Surgery, Monaldi Hospital, Azienda Ospedaliera dei Colli, Naples, Italy; 10 Department of General Surgery, ASST Nord Milano, Sesto San Giovanni Hospital, Milano, Italy; 11 Unit of General Surgery, Sassuolo Hospital, Azienda USL of Modena, Sassuolo, Modena Italy; 12 Department of Surgery, S. Salvatore Hospital, AST Marche 1, Pesaro e Fano, PU Italy; 13 Dipartimento di Chirurgia Generale e Specialistica, Chirurgia Generale, d’Urgenza e PS, Azienda Ospedaliera Universitaria Città Della Salute e Della Scienza di Torino, Turin, Italy; 14 Department of General, Emergency Surgery and New Technologies, Baggiovara General Hospital Azienda Ospedaliero Universitaria di Modena, Via Pietro Giardini 1355, Modena, 41126 Italy; 15 Department of General Surgery, ASST Nord Milano, Edoardo Bassini Hospital, Milano, Italy; 16 Department of Medicine, Surgery and Dentistry, University of Salerno, Salerno, Italy; 17 Gruppo Ospedaliero San Donato, University of Insubria, Milan, Italy; 18 Department of Surgery, Sapienza University, Rome, Italy; 19 Department of Surgery, S. Anna University Hospital and University of Ferrara, Ferrara, Italy; 20 Department of Surgical Sciences, University of Genoa, Genoa, Italy

**Keywords:** Stoma site incisional hernia, Biosynthetic mesh, Ileostomy closure, Quality of life, Prophylactic mesh, Surgical site complications

## Abstract

**Purpose:**

Incisional hernia after ileostomy closure is a complication that adversely affects patient outcomes, quality of life (QoL), healthcare resources, and related costs. Prophylactic mesh reinforcement, both biological and synthetic, has been shown to be safe and effective in preventing stoma site incisional hernia (SSIH). This study aimed to evaluate the use of a slowly absorbable biosynthetic mesh at the site of temporary ileostomy closure to prevent SSIH.

**Methods:**

This prospective, single-arm observational study was conducted across 14 Italian hospitals. Patients undergoing ileostomy reversal with retromuscular placement of a poly-4-hydroxybutyrate (Phasix™) mesh were enrolled. Endpoints included the incidence of radiologically and clinically detected SSIH at 1-year, postoperative morbidity, wound complications, and QoL assessed using the EuroQoL 5D-5 L and Carolinas Comfort Scale (CCS) questionnaires.

**Results:**

A total of 115 patients completed at least 1 year of follow-up and were included in the analysis. Seromas was the most frequent complication (8.6%). Superficial and deep SSIs occurred in 6% and 1.7% of patients, respectively. Three complications required intervention (2.6%), including one mesh removal. The 1-year SSIH rate was 1.7%, with a median follow-up of 477 d (range: 263–880). Considering the 55 patients (47.8%) who completed 2-year follow-up, the cumulative SSIH rate was 4.3%. EuroQoL 5D-5 L and CCS scores demonstrated progressive improvement in QoL and symptom reduction over time (*p* < 0.0001).

**Conclusion:**

These preliminary findings suggest that prophylactic placement of a slowly absorbable biosynthetic mesh (Phasix™) is safe and effective for SSIH prevention following ileostomy closure, without negatively impacting the patient’s QoL. The use of biosynthetic meshes may be considered a valid alternative to biological or synthetic meshes in this setting.

**Supplementary Information:**

The online version contains supplementary material available at 10.1007/s10029-025-03462-0.

## Introduction

A diverting loop ileostomy is commonly performed to protect high-risk anastomoses and reduce the severity of anastomotic leakage [1, 2]. Although these stomas are typically temporary, fecal diversion negatively impacts on patients’ quality of life [3, 4]. Consequently, a second surgery is usually planned 8–12 weeks after the primary procedure. However, stoma closure is associated with postoperative morbidity due to bowel and extraintestinal complications such as wound infection and stoma site incisional hernia (SSIH) [5, 6].

Systematic reviews have shown that the overall incidence of SSIH ranges from 0 to 35%, and approximately half of the affected patients require surgical repair [7, 8]. The actual incidence of SSIH following ileostomy closure is significantly influenced by study quality, endpoints, surgical technique, diagnostic method, and follow-up duration [9]. Thus, it is reasonable to assume that the problem is underdiagnosed or underestimated [10]. A retrospective review via medical records was conducted at Cleveland Clinic to evaluate the frequency of radiographic SSIH. CT-scans of 569 patients who underwent to diverting loop ileostomy reversal were assessed and approximately one-third of the developed SSIH within 1-year (36%) [11].

Compared with standard closure, prophylactic mesh reinforcement has been proven safe and effective in preventing SSIH [12–14]. It is not associated with higher incidence of wound infection, seroma and anastomotic leakage and need for a reintervention is significantly more frequent in no mesh group. Both nonabsorbable synthetic and biological prostheses with satisfactory outcomes have been described; however, the optimal type of mesh and its placement remain to be defined [15]. The risk of mesh infection in a contaminated field and the anatomy of the lateral abdominal wall play pivotal roles in SSIH prevention strategies.

The use of slowly absorbable biosynthetic mesh (Phasix™ BD) has gained increasing acceptance for the repair of complex and contaminated ventral hernias, with acceptable postoperative complication and recurrence rates [16–19]. These meshes offer a cost-effective alternative to biological meshes, showing good tolerance in contaminated fields and complete resorption within 12–18 months [20].

As no data currently exist on the use of slowly absorbable biosynthetic mesh for SSIH prevention, the aim of this study was to evaluate the performance of prophylactic Phasix™ mesh placement at the site of temporary ileostomy closure.

## Materials and methods

### Study design

This prospective, single-arm observational study was conducted across 14 Italian hospitals beginning in June 2020. The study was approved by the Institutional Review Boards of all participating centers and registered at www.clinicaltrials.gov (NCT05400083). Adult patients with primary benign or malignant disease scheduled for elective loop ileostomy reversal were eligible. Exclusion criteria included clinically relevant parastomal hernia requiring abdominal wall repair, incisional ventral hernia, and the need for laparotomy during ileostomy closure. All patients provided informed consent to participate in the study. All medical records were collected anonymously through a web registry (www.italianherniaclub.it) and accessible only to the coordinating center.

## Surgical procedure

Following peristomal skin incision, the bowel segments were mobilized and the abdominal cavity was carefully dissected. Bowel resection and anastomosis were performed at the surgeon’s discretion. The posterior rectus sheath was closed with continuous sutures. A poly-4-hydroxybutyrate (Phasix™ BD, Franklin Lakes, NJ) biosynthetic mesh was placed in a retromuscular (sublay) position with at least a 2-cm overlap and secured with four stitches. Phasix™ is a fully and slowly absorbable mesh indicated for soft tissue reinforcement, including in contaminated fields [21]. The anterior rectus sheath was then closed with continuous suturing, and skin closure was completed according to the surgeon’s preference.

## Endpoints and outcome measures

The primary endpoint was the incidence of radiologically detected SSIH at 1 year. In accordance with surveillance protocols, patients with colorectal cancer underwent a computed tomography (CT) scan 1 year following the primary surgery [22]; others underwent ultrasonography. Secondary endpoints included SSIH diagnosed by physical examination, postoperative morbidity, incidence of wound events, mesh-related complications, and quality of life (QoL) assessments.

Wound complications were classified as surgical site occurrences (SSOs), including surgical site infection (SSI), seroma, wound dehiscence, enterocutaneous fistula, wound cellulitis, non-healing incisional wound, fascial disruption, tissue ischemia or necrosis, serous or purulent drainage, stitch abscess, seroma, hematoma, and mesh infection or exposure. Events requiring procedural intervention (SSOPI) were defined as wound opening or debridement, suture excision, percutaneous drainage, or mesh removal [23]. Mesh-related complications included infection, displacement or removal.

Health-related QoL data was measured using the EuroQol 5D-5 L and Carolinas Comfort Scale (CCS) questionnaires at various follow-up intervals. The EuroQol 5D-5 L assesses five health dimensions: mobility, self-care, usual activities, pain/discomfort, and anxiety/depression. The utility weight, also known as the utility value or utility score, range from 0 (death-equivalent health state) to 1 (perfect health) [24], and was calculated using an algorithm for the Italian population [25]. The CCS questionnaire includes 23 items assessing health-related QoL after hernia mesh repair. It quantifies the severity of pain, mesh sensation, and movement limitation from the hernia or surgical site across eight daily activities: lying down, bending over, sitting up, performing activities of daily living, coughing or deep breathing, walking, climbing stairs, and exercising. The score for each item is recorded on a six-point Likert scale ranging from the absence of symptoms to disabling symptoms [26, 27]. Scores range from 0 to 115, with higher scores indicating worse QoL. Patients completed the questionnaires at 8 and 30-day and at 12 and 24 months postoperatively.

### Statistical analysis

Continuous variables were expressed as means ± standard deviations, and categorical variables were summarized as frequencies and percentages. Assuming a 5% 1-year incidence of radiologically detected hernia following prosthetic placement [12, 13], with 3% precision and 95% confidence, the calculated sample size calculated was 203 patients. Estimation of SSIH-free survival curve was done according to the Kaplan–Meier method.

Changes in QoL over time for both EuroQol 5D-5 L and CSS were analyzed using the Wilcoxon paired t-test comparing values at 8 d with those at later time points. A-value *p* < 0.05 was considered statistically significant. Given the exploratory nature of the analysis and the relatively small sample size, we opted not to impute missing QoL data to avoid introducing additional model uncertainty or bias associated with imputation methods. Instead, we conducted a complete case analysis, which provides a conservative estimate of outcomes and preserves the integrity of the observed data. Furthermore, the pattern and proportion of missing data were assessed and did not suggest systematic bias. Specifically, we analyzed at each time point all the questionnaires with complete responses and have reported the total number of cases analyzed for each QoL measure.

## Results

A total of 169 patients underwent ileostomy closure with prophylactic Phasix ™ mesh placement between June 2020 and December 2024. Among them, 115 patients completed at least 1 year of follow-up and were included in the analysis. Baseline characteristics and perioperative data are summarized in Table 1. The mean patient age was 68 years; 59% were male, and 37.4% were overweight. Temporary ileostomy was created during surgery in patients with colorectal cancer (73%), ovarian cancer (4.3%), or benign disease (22.6%). Although all patients received retromuscular Phasix™ mesh reinforcement, the skin was closed using purse-string or linear sutures in 36.5% and 58.2% of cases, respectively. A subcutaneous drain was used in 50 patients (43.5%).

**Table 1:** Baseline patients’ characteristics and perioperative details of patients who completed 1 year follow up. Body mass index (BMI), American society of anesthesiologists (ASA), chronic obstructive pulmonary disease (COPD), standard deviation (SD)

	*n* = 115 (%)
**Male (%)**	68 (59)
**Age (range)**	68 (23–87)
**BMI Kg/m**^**2**^ **(range)**	24 (16–33)
BMI Kg/m^2^ 25-29.9 (%)	43 (37.4)
BMI Kg/m^2^ ≥ 30 (%)	7 (6)
**ASA score**:	
ASA 1	18 (15.6)
ASA 2	53 (46)
ASA 3	43 (37.3)
ASA 4	1 (0.8)
**Risk factors**:	
Active Smoking	16 (13.9)
Previous abdominal wall infection	10 (8.7)
COPD	9 (7.8)
Diabetes mellitus	12 (10.4)
Immunodepression	2 (1.7)
**Primary disease**:	
Colorectal cancer	84 (73)
Ovarian cancer	5 (4.3)
Benign disease	26 (22.6)
**Side of ileostomy**:	
Right	110 (95.6)
Left	5 (4.4)
**Antibiotic prophylaxis (%)**	111 (96.5)
**Mean operative time (SD)**	109 (± 54)
**Adhesiolysis (%)**	64 (55.6)
**Skin closure**:	
Purse-string	42 (36.5)
Linear closure	67 (58.2)
Other	6 (5.2)
**Subcutaneous drain**	50 (43.5)

### Postoperative morbidity and SSIH

Postoperative complications are detailed in Table 2. The most common SSO was seroma (8.6%). Superficial and deep SSIs occurred in 6% and 1.7% of patients, respectively. Three complications (2.6%) required procedural intervention, including one mesh removal. Two of them were a man and a woman in their eighties who developed a subcutaneous collection that required drainage and antibiotic therapy. Both had linear skin closure with no drain. Finally, a 76-years old man required surgery and mesh removal because of abscess with purse-string suture dehiscence.

**Table 2:** Postoperative complications

	*n* = 115 (%)
**SSO (%)**	33 (28.6)
SSI - Superficial	7 (6)
SSI - Deep	2 (1.7)
Seroma	10 (8.6)
Hematoma	6 (5.2)
Wound dehiscence	5 (4.3)
Abscess	3 (2.6)
**SSOPI (%)**	3 (2.6)
Mesh removal	1 (0.8)
**Bowel occlusion**	0
**Median Hospital stay**,** days (range)**	5 (1–35)

**Fig. 1:**
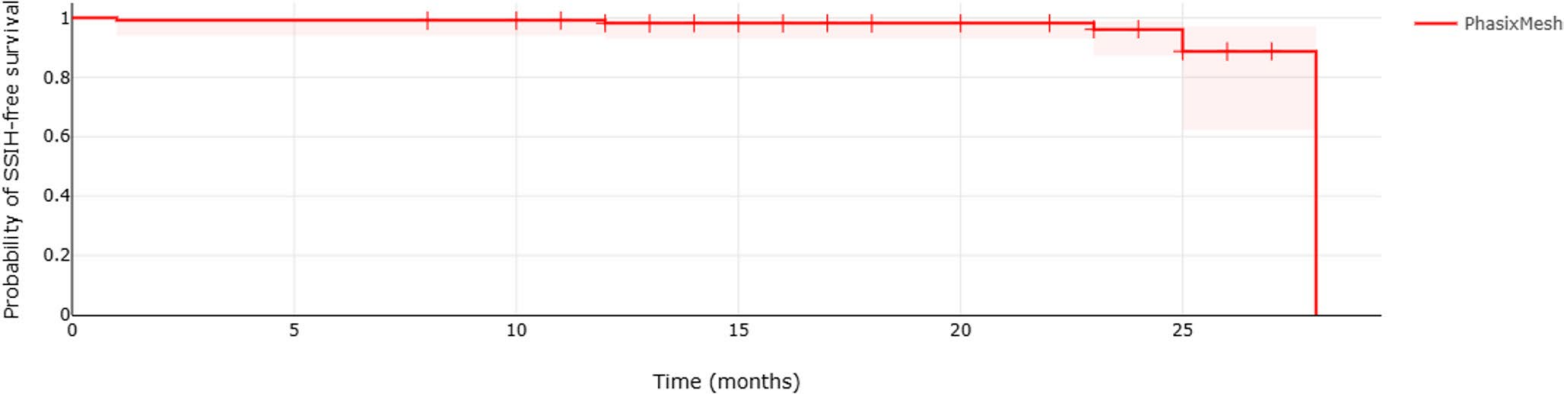
Estimated freedom of stoma site incisional hernia (SSIH) site curve with the Kaplan–Meier method of 115 patients (all patients completed 1-year of follow-up, 55 patients completed 2-years of follow-up)

A 1-year follow-up, SSIH at the prior ostomy site was detected in two patients (1.7%) based on physical and radiological examination (CT-scan and US, respectively). Considering the 55 patients (47.8%) who completed 2 years follow-up, the cumulative SSIH rate was 4.3% (Table 3) using CT-scan in all cases. The median follow-up period was 477 d (range: 263–880). The estimated freedom of SSIH curve according to the Kaplan–Meier method is shown in Fig. 1. Table 4 shows descriptive data of patients who developed SSIH.

**Table 3:** Stoma site incisional hernia (SSIH). * 55 patients completed 2-years of follow up

	*n* = 115 (%)
**SSIH (%) at 1 year**	2 (1.7)
Clinical and Radiologic	2 (1.7)
SSIH (%) at 2 years*	5 (4.3)
Clinical and Radiologic	4 (3.4)
Radiologic	1 (0.8)
**Median Follow up**,** days (range)**	477 (263–880)

**Table 4:** Details of patients who developed SSIH

	Patient #1	Patient #2	Patient #3	Patient #4	Patient #5
**Gender**	Female	Male	Female	Female	Female
**Age**	67	72	71	63	34
**BMI (Kg/m**^**2**^**)**	22	29	33	19	32
**Risk factors**:	Immunodepression Previous infection		Previous infection		Multiple abdominal surgery
**Malignant disease**	No	No	No	Yes	Yes
**Skin closure**	Purse-string	Linear closure with drain	Linear closure with drain	Purse-string	Linear closure with drain
**Wound complication**	SSI	Seroma	Seroma	No	Seroma
**Time to SSIH (months)**	1.1	12.2	23	25.5	28.8
**Method of diagnosis**	Clinical and Radiological	Clinical and Radiologic	Clinical and Radiologic	Radiologic	Clinical and Radiologic

## Quality of life assessment

Table 5 summarizes the QoL scores over time measured using the EuroQol 5D-5 L and CSS scales. Mean values, standard errors, and 95% confidence intervals (CIs) are reported for each time point, along with statistical significance (p-values).

**Table 5:** Changes in patients’ QoL over time for both EuroQol 5D-5 L and CSS (Carolinas comfort Scale)

QoL scale	Time	*n*	Mean value	Std. Err.	95% CI	*p*-value (8 days vs. other time points)
EuroQol 5D-5 L	8 days	110	0.884	0.012	0.861–0.908	-
	30 days	113	0.959	0.007	0.945–0.973	< 0.0001
	12 months	112	0.963	0.012	0.94–0.987	< 0.0001
	24 months	51	0.979	0.010	0.959-1.000	< 0.0001
CSS	8 days	62	14.887	2.023	10.843–18.932	-
	30 days	73	7.027	1.096	4.842–9.213	< 0.0001
	12 months	85	1.412	0.500	0.416–2.407	< 0.0001
	24 months	38	0.421	0.296	−0.179-1.021	< 0.0001

EuroQol 5D-5 L scores increased significantly from 0.884 at 8 d to 0.959 at 30 d (*p* < 0.0001), with further improvements at 12 months (0.963, *p* < 0.0001) and 24 months (0.979, *p* < 0.0001).

CSS scores showed a significant decrease from 14.887 at 8 d to 7.027 at 30 d (*p* < 0.0001), and further to 1.412 at 12 months (*p* < 0.0001) and 0.421 at 24 months (*p* < 0.0001).

## Discussion

This study presents preliminary findings on the safety and efficacy of prophylactic biosynthetic mesh placement for SSIH prevention after ileostomy reversal. Phasix™ mesh placed in the retromuscular position was effective in reinforcing the abdominal wall, with an SSIH rate of 1.7% at 1 year follow-up. Postoperative morbidity, including mesh-related complications, was acceptable, and QoL improved over time.

The true extent of SSIH remains debated. In a retrospective cohort study, De Keersmaecker et al. [28] reported a CT-detected SSIH rate of 11% in patients treated with temporary loop ileostomy reversal, with a mean follow-up period of 2.6 years. More recent studies report SSIH rates between 26% and 36% [12, 29, 30]. These differences may reflect variations in surgical techniques and materials, which strongly influence hernia risk [31]. Prophylactic mesh use is increasingly accepted as a to reduce incisional hernia after ileostomy closure [12–14, 32, 33], although the optimal mesh type and placement technique remain undetermined [15].

To the best of our knowledge, this is the first study demonstrating benefits of a slowly resorbable biosynthetic mesh for SSIH prevention at the ileostomy site. Pizza et al. [34] evaluated a faster-degrading (approximately 6 months) biosynthetic mesh (Bio-A, Gore) to prevent SSIH. The mesh was a copolymer of polyglycolic acid and trimethylene carbonate in a three-dimensional matrix, with a 1-year SSIH rate of 3.8%. In our study, the SSIH rate at 1 year was 1.7% after sublay Phasix™ mesh placement, which is lower than the rates reported for intraperitoneal (9%) and retromuscular (3.3%) biological meshes [12, 13]. Biological and biosynthetic prostheses are both used in contaminated surgical fields; however, biosynthetic meshes may be more favorable than biological ones due to better cost-effectiveness, high tensile strength, and lower complication rates. Furthermore, concerns have recently been raised regarding the long-term performance and cost-effectiveness of biological prostheses in preventing SSIH [35, 36].

Despite the theoretical infection risks in contaminated fields, synthetic non-resorbable meshes have demonstrated acceptable safety and feasibility when used for fascial closure reinforcement at ileostomy sites [14, 32, 33]. A multicenter randomized trial comparing synthetic and biological meshes in retromuscular sublay placement for incisional hernia prevention at the time of loop ileostomy closure found no differences in 30-d postoperative morbidity, patient-reported QoL, or 5-year SSIH rates between the two groups [37, 38]. Although there is increasing evidence of the safety of permanent mesh in a contaminated field [39–41], the risk of infectious complications remains a possible and feared event. Surgeons are reluctant to implant a permanent foreign material in contaminated fields during hernia surgery because of the increased risk of postoperative SSI and seroma, bowel adhesion, mesh erosion and expulsion, fistula formation and pain. This fear seems unjustified since the use of biologic mesh in patients undergoing ventral hernia repair results in more hernia recurrences and SSIs compared to non-absorbable mesh [42]. In this setting, the tolerance in contaminated surgical fields and the good performances of the biosynthetic mesh could mitigate the higher cost.

Concerning ileostomy reversal, there are a few and heterogeneous data. In the ROCSS study, wound infection rate was 16% in the biologic mesh group [12]. In contrast, Warren et al. reported 17% of SSO and 8% of SSI in patients who underwent to ileostomy closure with synthetic mesh [14]. Furthermore, compared with standard closure, ileostomy reversal with mesh positioning was not associated with higher risk of seroma formation [15]. Therefore, several factors may contribute to the development of wound complications included the surgical technique. The rational for choosing the retromuscular approach for prophylactic mesh reinforcement during stoma closure was based not only on the isolation of the mesh from the peritoneal cavity avoiding potential infection or adhesive disease but also because it allows the skin surrounding the purse-string closure to heal by secondary intention, unlike onlay mesh positioning. Furthermore, a recent meta-analysis demonstrated that the prophylactic placement of mesh in the retromuscular plane at the time of stoma closure is the most effective approach for reducing the incidence of SSIH and surgical site infection [43].

In our cohort, the SSO rate was 28.6%, including 7.8% with SSI and 8.6% with seromas. Only three patients (2.8%) required intervention, including one mesh removal. We speculate that wound complications were more likely associated with the high rate of linear skin closure (58.2%) rather than mesh placement. The purse-string technique has been associated with lower SSI rates and higher patient satisfaction than other techniques [44, 45]. Nonetheless, linear closure remains widely used, and ileostomy reversal techniques are not standardized [46].

Data on quality of life assessment are very limited in this context. Patient-reported outcomes using EuroQoL 5D–5 L and CCS questionnaires showed progressive QoL improvements and symptom reduction over time. EQ-5D-5 L scores reached 0.96 at 30 d, 0.96 at 1 year, and 0.98 at 2 years—higher than those reported in the ROCCS study [12] for both biologic and no mesh groups. For the EQ‑5D‑5 L, we referenced the instrument-defined minimal clinically important difference (MCID) estimates reported by McClure et al., which range from 0.037 to 0.069 across international value sets [47]. The utility gain observed in our analysis (0.095, calculated as 0.979–0.884) exceeds this established threshold, suggesting a clinically meaningful improvement in health status attributable to the intervention. Although a universally accepted MCID for the CCS has not yet been formally established, relevant benchmarks from the literature, considering a similar context, support the clinical relevance of our findings. In the recent GENESIS study, conducted across multiple European centers, Baig et al. reported a mean CCS score of 16.69 following inguinal hernia repair, reflecting the typical symptom burden in postoperative patients [48]. In comparison, our observed mean final CCS score of 0.421 indicates a markedly lower symptom load, pointing to a potentially substantial and meaningful improvement in postoperative comfort. These results reinforce the benefit of the evaluated strategy from both a generic and condition-specific patient-reported outcome perspective.

This study has several limitations. First, we presented preliminary results representing 56.6% of the calculated sample size and the findings should be interpreted with caution. Nevertheless, it is the largest study to date providing evidence-based outcomes on the use of biosynthetic mesh at the ileostomy closure site with a 12-month follow-up. Second, the absence of a control group limits the ability to compare outcomes with alternative treatment options. Third, given that the resorption time of the prosthesis is estimated to be 12–18 months, incisional hernias may develop after the 12-month mark. Therefore, follow-up data at 24 months have also been included. Fourth, the clinical characteristics of the patients varied within the study population, which may have affected the consistency of long-term health improvements. However, the cohort was representative of the surgical practices commonly used in most hospitals. Finally, in this study we combined CT-scan and ultrasonography. This is a further limitation because difference in sensibility and specificity between the imaging techniques exists. Although CT-scan is preferred for identifying recurrence, a patient-tailored imaging technique is advisable [49].

## Conclusions

Preliminary findings from this study demonstrated low rates of incisional hernia and postoperative wound complications in patients undergoing ileostomy reversal with slowly absorbable biosynthetic mesh reinforcement, without compromising QoL. These results suggest that Phasix™ mesh may be a safe and effective for SSIH prevention in contaminated surgical fields. However, no firm conclusions can be drawn and further confirmation is needed by completing follow-up and expanding the sample size. In addition, future investigations are warranted to compare benefits and drawbacks of synthetic and biosynthetic prostheses in this setting.

## Supplementary Information

Below is the link to the electronic supplementary material.

ESM 1 (DOCX 34.0 KB)

## Data Availability

The data that support the findings of this study are available from the corresponding author upon reasonable request. All authors approved the final version and meet authorship criteria.
